# Calcitonin gene-related peptide facilitates sensitization of the vestibular nucleus in a rat model of chronic migraine

**DOI:** 10.1186/s10194-020-01145-y

**Published:** 2020-06-10

**Authors:** Yun Zhang, Yixin Zhang, Ke Tian, Yunfeng Wang, Xiaoping Fan, Qi Pan, Guangcheng Qin, Dunke Zhang, Lixue Chen, Jiying Zhou

**Affiliations:** 1 Department of Neurology, The First Affiliated Hospital of Chongqing Medical University, 1st Youyi Road, Yuzhong District, Chongqing, 400016 China; 2 Department of Vascular Surgery, The First Affiliated Hospital of Chongqing Medical University, Chongqing, China; 3 Laboratory Research Center, The First Affiliated Hospital of Chongqing Medical University, Chongqing, China

**Keywords:** CGRP, Vestibular migraine, Trigeminal nucleus caudalis, Vestibular nucleus, Central sensitization, Anti-CGRP treatment

## Abstract

**Background:**

Vestibular migraine has recently been recognized as a novel subtype of migraine. However, the mechanism that relate vestibular symptoms to migraine had not been well elucidated. Thus, the present study investigated vestibular dysfunction in a rat model of chronic migraine (CM), and to dissect potential mechanisms between migraine and vertigo.

**Methods:**

Rats subjected to recurrent intermittent administration of nitroglycerin (NTG) were used as the CM model. Migraine- and vestibular-related behaviors were analyzed. Immunofluorescent analyses and quantitative real-time polymerase chain reaction were employed to detect expressions of c-fos and calcitonin gene-related peptide (CGRP) in the trigeminal nucleus caudalis (TNC) and vestibular nucleus (VN). Morphological changes of vestibular afferent terminals was determined under transmission electron microscopy. FluoroGold (FG) and CTB-555 were selected as retrograde tracers and injected into the VN and TNC, respectively. Lentiviral vectors comprising CGRP short hairpin RNA (LV-CGRP) was injected into the trigeminal ganglion.

**Results:**

CM led to persistent thermal hyperalgesia, spontaneous facial pain, and prominent vestibular dysfunction, accompanied by the upregulation of c-fos labeling neurons and CGRP immunoreactivity in the TNC (c-fos: vehicle vs. CM = 2.9 ± 0.6 vs. 45.5 ± 3.4; CGRP OD: vehicle vs. CM = 0.1 ± 0.0 vs. 0.2 ± 0.0) and VN (c-fos: vehicle vs. CM = 2.3 ± 0.8 vs. 54.0 ± 2.1; CGRP mRNA: vehicle vs. CM = 1.0 ± 0.1 vs. 2.4 ± 0.1). Furthermore, FG-positive neurons was accumulated in the superficial layer of the TNC, and the number of c-fos+/FG+ neurons were significantly increased in rats with CM compared to the vehicle group (vehicle vs. CM = 25.3 ± 2.2 vs. 83.9 ± 3.0). Meanwhile, CTB-555+ neurons dispersed throughout the VN. The structure of vestibular afferent terminals was less pronounced after CM compared with the peripheral vestibular dysfunction model. In vivo knockdown of CGRP in the trigeminal ganglion significantly reduced the number of c-fos labeling neurons (LV-CGRP vs. LV-NC = 9.9 ± 3.0 vs. 60.0 ± 4.5) and CGRP mRNA (LV-CGRP vs. LV-NC = 1.0 ± 0.1 vs. 2.1 ± 0.2) in the VN, further attenuating vestibular dysfunction after CM.

**Conclusions:**

These data demonstrates the possibility of sensitization of vestibular nucleus neurons to impair vestibular function after CM, and anti-CGRP treatment to restore vestibular dysfunction in patients with CM.

## Introduction

Migraine associated with recurrent vestibular symptoms are no strangers to clinicians. Migraine has been estimated to affect about 15% of the general population [1], and up to 85% patients with migraine suffers from balance problems and dizziness [2, 3]. Recently, International Headache Society and Bárány Society have prompted efforts to recognize and classify vestibular migraine (VM), which incorporates vestibular and migraine symptoms, as a novel subtype of migraine in International Headache Classification of Headache Disorders, 3rd edition [4]. Whether vestibular symptoms are associated symptoms as photophobia and phonophobia in migraine, or the distinctive entity that correlates with migraine are still in debate [5].

Migraine has been proposed as a complex sensory disorder with abnormal sensitization in central trigeminal-vascular system [6, 7]. Central sensitization of trigeminovascular neurons in trigeminal nucleus caudalis (TNC) has been considered to be the neural basis for sensory hypersensitivity in migraine, such as tactile allodynia [6]. We previously showed that the development of vertigo was lagged behind migraine by an average of 6 years in VM [7, 8], and the prevalence of vertigo prominently increased in chronic migraine (CM) compared with episodic migraine [9]. Whether vestibular symptom is one of clinical manifestations of central sensitization for migraine remains unproven. Clinical data demonstrated that patients with VM exhibited abnormally elevated vestibular-ocular threshold [10], as well as reduced roll tilt threshold [11] compared to migraine patients without vestibular symptoms and patients with a peripheral inner-ear disorder, indicating that dysfunction of the vestibular nuclei (VN) might be an underlying mechanism for vestibular symptoms in VM. As an important sensorimotor center in brainstem, VN and its direct connection with TNC had been demonstrated in rodents [12]. Further, nociceptive trigeminal stimulation could induce vertigo in patients with migraine rather than controls [13]. Above data suggested that migraine-mediated sensitization of the TNC might affect the sensitivity of VN which receives TNC projections. However, whether vestibular sensitization is the potential mechanism for vestibular symptoms in migraine remains largely unknown.

The neuropeptide calcitonin gene-related peptide (CGRP) plays a crucial role in migraine based on its efficacy of anti-CGRP treatment in clinical trials against headache, and CGRP administration triggers delayed migraine-like headache in patients with migraine [6, 14]. Pharmacological and electrophysiological studies demonstrated that CGRP might facilitate nociceptive signals in the spinal cord, rather than produce nociception, which contributes to the development of central sensitization [15]. Our previous study also found a significantly increased expression of CGRP in TNC in a rat model of CM, and that was parallel to the development of central sensitization [16]. Meanwhile, rodents exposed to repeated bouts of rotary stimulation had an increased number of CGRP expressing neurons in VN, whilst anisodamine could significantly decrease the CGRP expression in VN [17], pointing that CGRP might be a mediator in trigeminal-mediated sensitization of VN.

Here our aim was to determine whether recurrent nitroglycerine (NTG) administration could induce vestibular dysfunction that is associated with migraine, and subsequently the effectiveness of CGRP knockdown to relieve these symptoms. We also wanted to determine central sensitization in TNC as a precursor in migraine-associated vestibular dysfunction. Using retrograde labeling of central trigeminocervical and vestibular neurons, we analyzed the functional connection changes caused by recurrent NTG administration and whether this manifested as a potential neural basis of VM in a preclinical model.

## Materials and methods

### Animals

Male Sprague Dawley rats (weighting 250–350 g) obtained from the Experimental Animal Center of Chongqing Medical University (Chongqing, China) were used in accordance with international standards under protocols approved by the Commission of Chongqing Medical University for ethics of experiments on animals. All studies were complied with the ARRIVE guidelines. All animals were randomly assigned to experimental groups. Animals were housed on a 12 h light-dark cycle with access to food and water ad libitum.

### Chronic migraine (CM) model

The formula of NTG for injection was prepared as described previously [16, 18]. In brief, a stock solution of 5.0 mg/ml NTG (Beijing Regent, China) was dissolved in 30% alcohol, 30% propylene glycol, and distilled water. Prior to each administration, NTG was diluted to 1 mg/ml. Animals randomly received intraperitoneal administration of NTG (2 ml/kg) or vehicle (0.9% saline) every 2 days for 9 days [16]. According to previous study, 0.9% saline was chosen as vehicle control due to the similar mechanical thresholds between 0.9% saline and 6% propylene glycol + 6% alcohol + 0.9% saline [16].

### Peripheral vestibular dysfunction model

Single trans-tympanic injection of kainic acid (12.5 mM, 100 μL) was performed under anesthesia according to the procedures previously described [19, 20]. Briefly, rats were anesthetized with 10% chloral hydrate (4 ml/kg, intraperitoneal) combined with analgesics (0.01 mg/kg buprenorphine) as described previously [21], and then kept in lateral position. A sterile insulin syringe (0.5 × 16 mm) was applied to penetrate the posterior portion of the tympanic membrane, and either kainic acid or 0.9% saline was injected into the right side of middle ear cavity slowly. After injection, the animal was kept in lateral position by resting on a electronical heating pad until recovered from the anesthesia.

### Stereotaxic surgery procedures and intracranial injection procedures

Seven days prior to the first NTG or saline injection, rats were deeply anesthetized as described in Peripheral vestibular dysfunction model and placed on a stereotaxic frame for the injection of the retrograde tracer fluorogold (FG) or Cholera Toxin B (CTB) -555. At the end of the procedure, rats were treated as described in Peripheral vestibular dysfunction model for recovery.

A 2% solution of FG (Fluorochrome LLC, USA) was injected in bilateral caudal ventrolateral medullary regions following the coordinates as described in previous study (mediolateral: ±22.0; anteroposterior: − 12.8; dorsoventral: − 10.0) [22]. For TNC injection, CTB-555 (C334776, Invitrogen) was positioned at 1–2.4 mm caudal to the obex and inserted into the bilateral caudal spinal trigeminal interpolaris (Sp5ic), as lateral (about 2.7 mm) as possible according to the previous studies [23, 24]. A volume of 1 μl per site was slowly injected (over 30 s) via Hamilton microsyringe (10 μL), and the needle was left in place for additional 5 min when completed the injection.

### Lentiviral vectors preparation and trigeminal injection procedure

The recombinant lentivirus containing the rat calca gene RNA interference (RNAi) sequence (sense: TGGAGCAGGAGGAGGAACA) was packaged using hU6-MCS-Ubiquitin-EGFP-IRES-puromycin vector by GeneChem (Shanghai, China). Blank lentivirus without RNAi sequence was prepared as control.

The lentivirus (8 × 105 TU, 1 μL) was injected into the trigeminal ganglion as described previously [25]. After deeply anesthetized as described in Peripheral vestibular dysfunction model, rats were kept in lateral position on a heating pad. Facial hair between the ear and eye was shaved to expose the injection site (between the tympanic bulla, condylar process and angular process). The direction of injection was 90° to the head midline and 15° to the coronal plane. The injection depth of needle was 9 mm. Rats randomly received either 10 μL of viral vectors containing CGRP RNAi or blank viral vectors via microsyringe, and the needle was left in place for additional 5 min when completed the injection.

### Behavioral tests

Behavioral assessments were performed by an investigator blinded to experiments as previously described [16]. Basal responses of thermal hyperalgesia was performed 15–20 min before each NTG injection. Post-treatment responses of thermal hyperalgesia, head grooming, balance beam walk, and negative geotaxis were performed at 2 h after each NTG administration. Vestibular dysfunction scores were evaluated at 2 h after the fifth NTG or single kainic acid injection [26–28]. Thermal hyperalgesia and head grooming were considered as indexes of spontaneous cutaneous cephalic and extracephalic allodynia [29]. Balance beam walk, negative geotaxis and vestibular dysfunction were a series of behavioral assessments for rodents’ vestibular system [26–28, 30, 31].

#### Thermal hyperalgesia

Thermal sensitivity was tested using Hargreaves radiant heat apparatus (model PL-200, IITC, Taimeng, Chengdu, China) [21, 32]. Briefly, rats were accustomed in a smooth, glass-floored transparent cage (height: 15 cm, width: 20 cm and length: 20 cm) for 30 min. Infrared radiation (intensity: 20) was then applied to the center of rats’ hind paws. Withdrawal latency was automatically recorded when the hind paw moved. Thermal stimulation was stopped after 20 s in case of tissue damage. Each rat was tested three times with an interval of 5 min of each stimulation to calculate the average latency.

#### Head grooming

After each NTG injection, animals were placed in Plexiglas cages to habituate for 20 min as described previously [29, 33]. The time that animals spent on head grooming was recorded by a video camera, and the total recording time for per rat was 20 min.

#### Balance beam walk

As described previously, a balance beam (length: 190 cm, diameter: 2.5 cm) was placed horizontally with an elevation of 40°, and a cushion was placed below to protect animals that fell [30]. A black plastic box (13 × 22 cm with a 5 × 6 cm doorway) was set at the upper end of the rod to motivate the animal to cross the beam. Previous study demonstrated that rodents naturally sought out the darkness and run in an upwards direction [31]. The duration for traversing was recorded (3 trials per rat). Before each trial, animals were allowed to have a rest for 90 s.

#### Negative geotaxis

Rats were placed onto a 40° slope with their head downward as described previously [30), and the duration for a turn of 180° was recorded. The maximum recording time was 20 s (3 trials per rat). The value of all three trials were averaged.

#### Vestibular dysfunction scores

As previously described, animal scores for vestibular functions evaluated the following four parameters: dyskinetic head movements, circling, retro-pulsion, tail-hang reflex, contact-inhibition reflex, and air-righting reflex [19, 26–28]. Each parameter was rated from 0 (normal behavior) to 2 (severe response). A maximum score of 12 was given with higher scores indicating worse performance.

1. *Dyskinetic head movements and circling*

These experiments contained two parts sharing same scoring system. Rats were placed individually in a transparent chamber (50 × 50 cm), and were observed for head weaving or circling for a period of 2 min. The response was rated as follows: 0 = neither dyskinetic head movements nor circling, 1 = sporadic dyskinetic head movements or circling, and 2 = frequent to persistent dyskinetic head movements or circling.

2) *Retro-pulsion*

Rats were also placed individually in a transparent chamber as described above for an observation period for 2 min. The response was rated as follows: 0 = no backward steps; 1 = few backward steps; 2 = persistent backward walk.

3) *Tail-hang reflex*

Rats were lifted by the tail and the response was rated as follows: 0 = straight body posture towards the ground with extension of forelimbs, 1 = sporadic bending the body ventrally, and 2 = persistently bending the body towards its tail.

4) *Contact inhibition of righting reflex*

The rats were placed in a supine position while its back maintained contact with the table. Another horizontal surface was placed to touch its feet. The ability to go from a supine to a prone position was rated as follows: 0 = completely righting, 1 = partial righting, and 2 = failed in righting.

5) *Air righting reflex*

The rats were held in a supine position and dropped from a height of 30 cm onto a foam cushion. The response was graded as follows: 0 = completely righting and landing squarely on their feet, 1 = partial righting or landing on side, and 2 = failed in righting and landing on back.

### Quantitative real-time polymerase chain reaction (qPCR)

Based on previous studies, the rat TNC tissues, localized between − 14 and − 16 mm from bregma, and the VN tissues, localized between − 10 and − 12 mm from bregma, were extracted based on the rat brain atlas of Paxinos and Waston (6th edition) [16, 30]. Total RNA from tissues was extracted using RNAiso Plus reagent (TaKaRa, Dalian) (21). RNA concentration and purity were quantified spectrophotometrically with NanoDrop (Thermo, USA). First-strand cDNA was generated using1 mg of total RNA with reverse transcriptase PrimeScript™ RT Reagent Kit (Takara, Dalian). The mRNA expression of rat CGRP was detected by qPCR withSYBR® Premix Ex TaqTM II (TaKaRa, Dalian) with a CFX96 Touch thermocycler (Bio-Rad, USA) according to the manufacturer’s recommendation. The reaction mixture (20 μL total) consisted of 10 μL 2× SYBR® mix, 8 μL nuclease-free water, 0.5 μL of primers (rat CGRP: F: 5′-GTTGGCTATTGTGCATCGTGTT-3′, R: CCGCTTGAGGTTTAGCAGAGTTA-3′; GAPDH, F: 5′-ATGACTCTACCCACGGCAAGCT-3′, R: 5′-GGATGCAGGGATGATGTTCT-3′) and 1 μL diluted cDNA. The reactions were performed as follows: an initial 5 min’s denaturation step, followed by 35 cycles of 95 °C for 30 s, 63 °C for 30 s, and 72 °C for 45 s, as well as a 7 min final extension step. Relative mRNA levels were calculated using the ΔΔCq method using GADPH mRNA as an internal control.

### Histological analysis

Rats were deeply anesthetized with 10% chloral hydrate (4 ml/kg, intraperitoneal) and subcutaneously injected with 0.01 mg/kg buprenorphine, then transcardially perfused with heparinized 0.9% saline followed by 4% paraformaldehyde (PFA) in 0.1 M PBS (pH 7.4) (for immunofluorescence staining) or 2.5% glutaraldehyde (for transmission electron microscopy).

#### Immunofluorescence staining

Immunofluorescence staining was performed on fixed frozen brain sections as previously reported [16]. Brains were post-fixed in 4% PFA overnight at 4 °C, and cryoprotected at 4 °C by successively immersed in a gradually increased concentration of sucrose solution (20% to 30%) until the tissues sank to the bottom. The transverse and sagittal sections of the VN, as well as the transverse sections of TNC were cut at 10 μm thickness on cryostat (Leica, Japan). Sections were permeabilized with 0.3% Triton X-100 (Beyotime, China) for 10 min at 37 °C, then blocked in 10% normal goat serum (Boster, China) in PBS for 30 min. After washing with PBS for three times (5 min each), brain sections were incubated at 4 °C overnight with the following primary antibodies: anti-CGRP (1:100, sc-57,053, Santa Cruz, USA); anti-c-fos (1:1000, 226,003, SYnaptic Systems, Germany); anti-NeuN (1:100, ab104224, Abcam, UK); anti-Glutamate (1:100, G9282, Sigma, USA); anti-Glutamic Acid Decarboxylase 65/67 (GAD67, 1:100, G5163, Sigma, USA). Sections were rinsed three times with PBS, followed by a 90 min’s incubation at 37 °C with secondary antibodies: Alexa Fluor 488-conjugated goat anti-rabbit immunoglobulin G (IgG, 1:200, Beyotime, China), Alexa Fluor 488-conjugated goat anti-mouse IgG (1:200, Beyotime, China), and Cy3-conjugated goat anti-mouse IgG (1:200, Beyotime, China), and then washed again with PBS for three times. Finally, slides were covered with DAPI (Beyotime, China). The sections were imaged under a fluorescence confocal microscope (ZEISS, Germany).

Sections were examined and images were acquired with a ZEISS Axio Imager A2 microscope equipped with structured illumination (Zen). Morphological identification of TNC was determined as previously described [16, 21]. The VN was identified under light microscopy through Paxinos and Watson’s stereotaxic atlas. C-fos+, CGRP+, and FG+ cells were quantified on both sides for TNC and VN from selected serial transverse sections collected from the rostral to caudal part of the brainstem. The number of immunolabeled cells was counted through the optical fractionator method under a 20x objective [34]. Each rat was collected six sections from the TNC and another six sections from the VN for cell counting, and the results were averaged and expressed as cells/field of view. To avoid counting the same neuron twice, each section was separated by at least 250 μm [22]. For each labeling in the TNC and VN, two quantified images were randomly selected with at least 480 μm apart [35]. Considering that CGRP exclusively expressed on the terminals of primary afferent fibers in the TNC, the mean optical density (OD) of CGRP were used to estimate the fluorescence intensity of CGRP over a microscopic field of 20×. Two investigators were counted individually, and both of them were blinded to the groups.

The proportion of FG-filled TNC and CTB-555-filled neurons that also contained c-fos labeling was analyzed as described above. FG-positive/c-fos-positive and FG-positive/c-fos-negative cells were counted at × 20 magnification. The OD of CTB-555 in the VN were analyzed under × 10 magnification.

#### Transmission electron microscopy

Tissue preparation for transmission electron microscopy was performed as previously described [19, 36]. In brief, Inner ears were rapidly removed, followed by unfolding acoustic vesicle to expose cochlea and its surrounding structures. Whole vestibular organs were fixed by 4% glutaraldehyde with phosphate buffer (0.1 M, PH 7.2) at 4 °C overnight. Then, vestibular organs were decalcified in 10% EDTA for 14 d at room temperature. After being rinsed with phosphate buffer for 30 min, the samples were post-fixed with 1% osmium tetroxide (OsO4) for 1.5 h, followed by staining with aqueous uranyl acetate for 1 h, dehydrating in a graded series of ethanol and embedding in Epon-Araldite resin (Canemco & Marivac, Lakefield, Quebec, Canada). Ultrathin sections (silver-gold, 80–90 nm) were cut using an ultramicrotome (Reichert-Jung, Inc., Cambridge, UK), and sections were counterstained with 0.3% lead citrate, and visualized on a transmission electron microscope (EM420, Koninklijke Philips Electronics N.V., Amsterdam, The Netherlands).

Cells were classified based on morphological criteria in accordance with previous protocol [19, 20]. The proportion of hair cells contacted by their swollen terminals (width > 1 μm) were summed on 3 ultrathin sections collected from 3 different sets from striolar and extrastriolar regions. These images were analyzed using Image-pro Plus 6.2 software (Bethesda, MD, USA).

### Statistical analysis

Data are presented as mean ± SEM. All statistical analyses were performed by SPSS software (SPSS Inc., IBM, USA). Student’s *t*-test was used for statistical comparisons between two groups. One-way or two-way ANOVA followed by post hoc analysis with the Tukey test was used for statistical comparisons among groups. Mann-Whitney *U* test was selected for the nonparametric analysis. Results were defined significant at *p* < 0.05.

## Results

### Recurrent NTG injection induced hyperalgesia and vestibular dysfunction

**Fig. 1:**
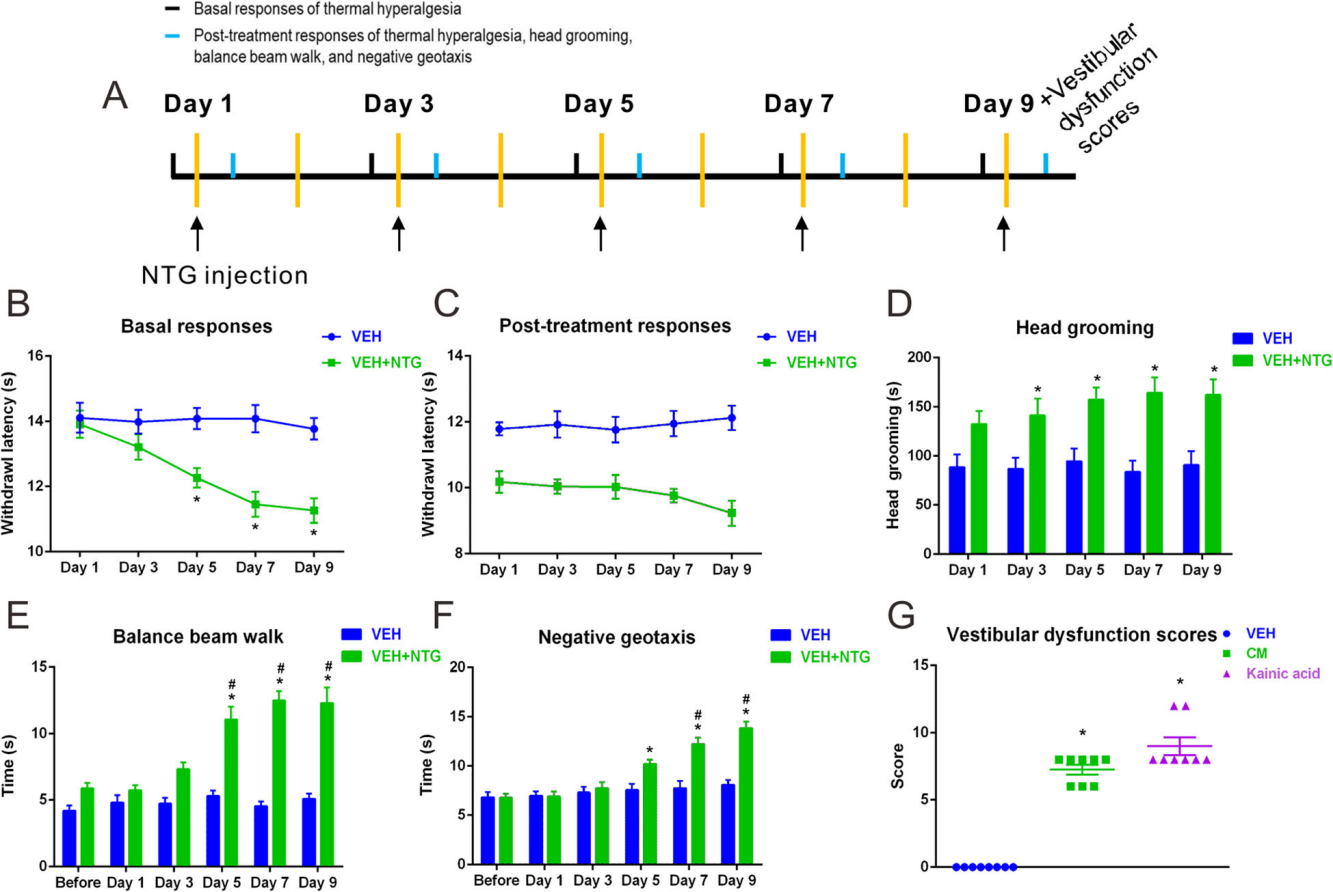
Recurrent nitroglycerine (NTG) injection induced thermal hyperalgesia and vestibular dysfunction. **a** Timeline of behavior studies’ protocol. **b** Basal and post-treatment responses (**c**) of hindpaw thermal hyperalgesia were markedly increased in a time dependent manner after NTG injection. **d** Head grooming time was significantly increased in NTG-treated rats compared with vehicle-treated group. Balance beam walk (**e**), geotaxis reflex (**f**) and vestibular dysfunction scores (**g**) showing that repeated NTG administration produced significant impairments in dynamic and static postural control compared with the vehicle group. *n* = 8/group. Data are mean ± SEM. Analysis of variance (ANOVA), Tukey; *p < 0.05 compared with vehicle, #*p* < 0.05 compared with before NTG injection

Consistent with our previous studies [37], we observed that in rats treated with NTG, the paw withdrawal latencies to noxious heat were markedly decreased in a time dependent manner on day 5, 7, and 9 as compared to vehicle control group (Fig. 1b). Chronic injection of NTG produced progressive basal hypersensitivity (Fig. 1b) and acute allodynia (Fig. 1c). We also found that NTG significantly increased head grooming time on day 3, 5, 7, and 9 as compared to vehicle control group (Fig. 1d).

We then asked whether chronic intermittent injection of NTG exacerbated vestibular functions. The alternation of vestibular function was determined by a series of behavior studies: balance beam and negative geotaxis for the assessment of dynamic postural changes during vestibulospinal reflexes; vestibular dysfunction scores for the evaluation of rodents’ static postural control [26–28]. NTG injection extended the time that rats spent traversing the balance beam, reaching significantly on day 5, 7, and 9 as compared to the vehicle and before injection data (Fig. 1e). On day 9, NTG-received rats needed approximately triple time to traverse the balance beam as compared to the vehicle (Fig. 1e). Similar results were observed in negative geotaxis test. NTG-received rats spent significantly more time to turn to 180° upward as compared to the vehicle on day 5, 7, and 9 (Fig. 1f). Furthermore, the severity of static vestibular dysfunction was significantly increased in NTG-received rats compared with the vehicle (Fig. 1g). Behavior studies revealed that the development of vestibular dysfunction was in parallel with that of pain hypersensitivity after CM.

### Morphology of vestibular afferent terminals was preserved after CM

Among patients with migraine, up to 25% prevalence of peripheral vestibular abnormities has been reported, including peripheral nystagmus and unilateral hearing loss [38, 39]. In the present study, vestibular dysfunction results showed that the severity of CM-induced vestibular dysfunction was comparable with the kainic acid treated group (Fig. 1g). Thus, researchers postulated that the damage of vestibular afferent terminals caused by migraine-associated vasospasm might mediate vestibular symptoms [40–42]. To investigate whether the CM-induced vestibular dysfunction stem from direct damage to vestibular apparatus, we used electron microscopy to examine the percentage of type I hair cells displaying swollen terminals in rat right utricles 2 h post CM. Since the severity of vestibular dysfunction and glutamate-elicited tissue damage peaked at 2 h post injection, morphological evaluation at subcellular level were determined at 2 h post kainic acid injection [19].

**Fig. 2:**
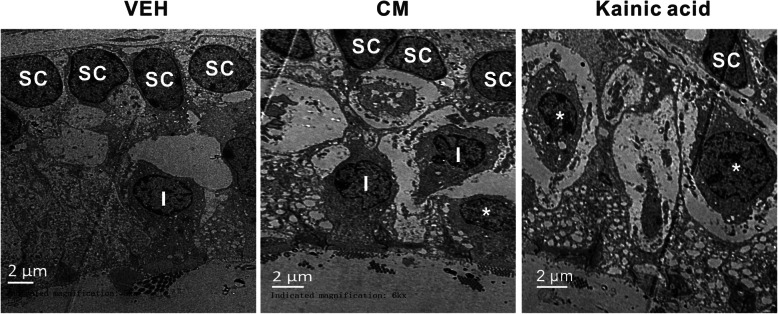
Morphological evaluation of excitotoxic damages by electron microscopy. In vehicle tissue, type I hair cells (I) surrounded by calyx nerve endings and supporting cells (sc) were in order. In CM tissue, supporting cells (sc) were still in order, but type I hair cells (I) were separated by vacuoles. In injured inner ear, most hair cell type became undetermined (asterisk) and numerous swollen nerve terminals below hair cells were observed. Scale bars = 2 μm, *n* = 3/ group

In vehicle-treated group, the typical morphology of sensory epithelia, that supporting cells were in order and type I hair cells were like pear shape surrounded by calyx terminals, was observed (Fig. 2). CM group’s supporting cells were still present in order, but hair cells were partially separated by few vacuoles (Fig. 2). Following kainic acid injection, supporting cells were not in order and the type of hair cells became undetermined that the afferent terminals were highly swollen leaving large vacuoles among the sensory epithelia (Fig. 2). The population of hair cells displaying swollen terminals in kainic acid-treated group (71.9 ± 4.1%) was significantly higher (*p* < 0.05) than those observed in vehicle (5.1 ± 1.4%) and CM group (9.2 ± 1.3%), while the proportion was comparable between vehicle and CM group. Preserved morphology of vestibular end-organs in CM group indicated that CM-induced vestibular dysfunction might likely to be central origin.

### Elevation of c-fos and CGRP in the TNC and VN after CM

**Fig. 3:**
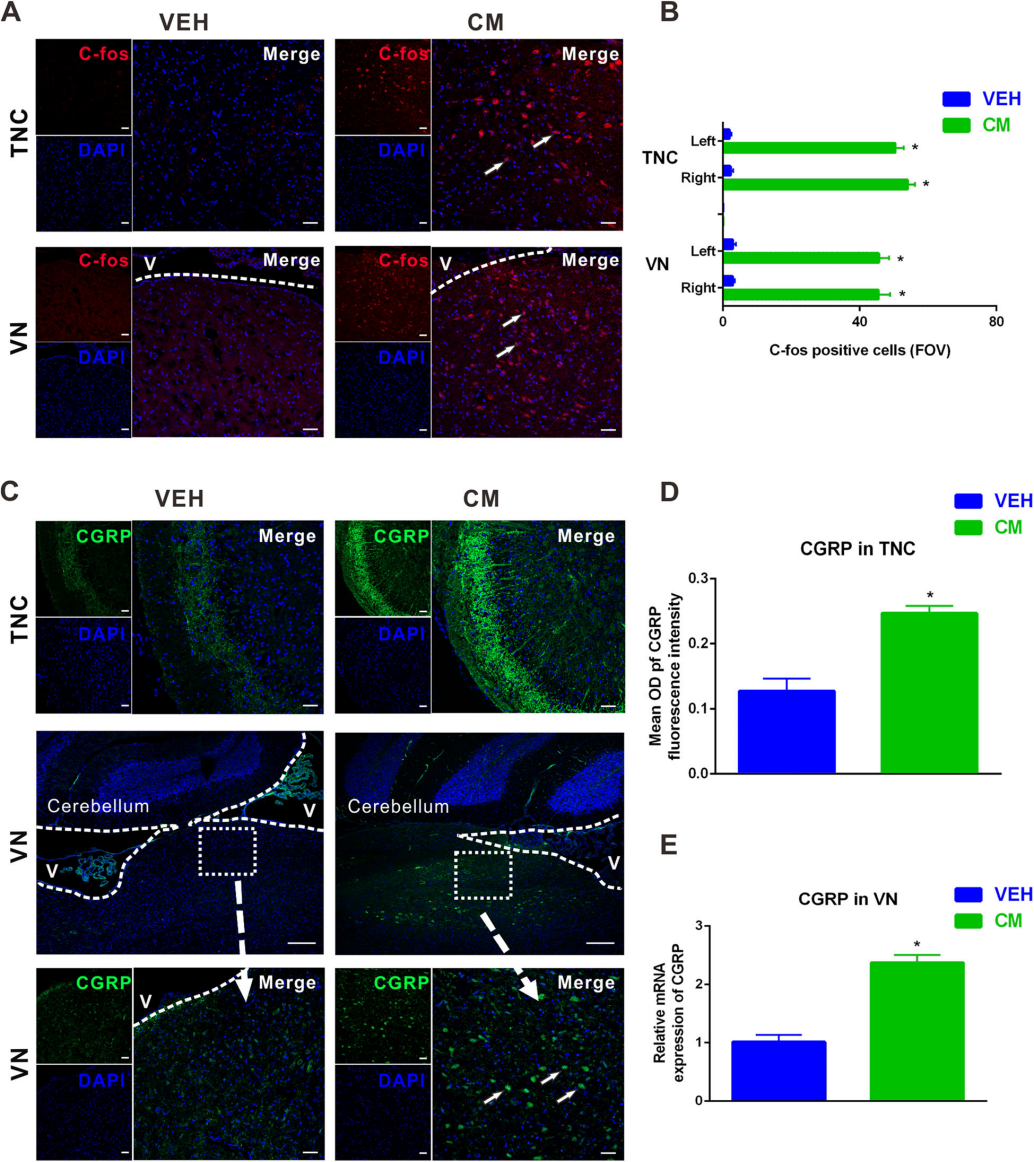
Upregulation of c-fos and calcitonin gene-related peptide (CGRP) in the trigeminal nucleus caudalis (TNC) and vestibular nuclei (VN) after CM. **a** Representative images of c-fos (red)-positive staining in the TNC and VN post CM. **b** Quantification of c-fos + cells in right and left sides of TNC and VN, showing significantly increased number of c-fos + cells after CM compared with vehicle group. FOV = 1.02 × 10^6^ μm^3^. **c** Representative images of CGRP (green)-positive staining in the TNC (upper panel) and VN (middle and lower panel) after CM. Data showed that CM group had greater CGRP signals (**d**) in the TNC compared with vehicle group. **e** qRT-PCR results showed that the mRNA level of endogenous CGRP significantly increased after CM. **a** (**c**: upper and lower panel) Scale bar = 50 μm, (**c**: middle panel) Scale bar = 100 μm; *P < 0.05 compared with vehicle, *n* = 8/group. Data are mean ± SEM. Student’s *t*-test. V: ventricle

C-fos has been considered to be a marker for the neuronal activation after noxious stimulation, and its upregulation underly the sustained mechanical hypersensitivity in CM [37, 43]. Immunofluorescence staining showed that the number of c-fos positive cells in the TNC was significantly increased post CM (vehicle vs. CM = 2.9 ± 0.6 vs. 45.5 ± 3.4, Fig. 3a and b). Given the key position of VN in vestibulospinal reflexes and posture control [30], we next sought to examine the changes of VN neurons after CM. Accordingly, c-fos immunoreactivity was also significantly increased in the VN post CM (vehicle vs. CM = 2.3 ± 0.8 vs. 54.0 ± 2.1, Fig. 3a and b). No evident difference was detected in the overall number of c-fos positive cells between right and left side of the TNC and VN (Fig. 3b).

Moreover, CGRP emerges as a critical endogenous mediator of migraine, especially in the development and maintenance of central sensitization [37, 44]. The density of CGRP-immunoreactive fibers in the TNC was significantly increased in the superficial layers of TNC after CM (vehicle vs. CM = 0.1 ± 0.0 vs. 0.2 ± 0.0, Fig. 3c and d). We further suspected whether endogenous CGRP expression in the VN would be changed after NTG administration. We analyzed CGRP expression in the VN by qPCR after CM. The mRNA level of CGRP was significantly increased after CM relative to vehicle-treated group (Fig. 3e). Although CGRP positive cells were widely distributed throughout the four major vestibular nucleus as defined in the rat brain atlas of Paxinos and Watson, we found that CGRP immunoreactivity was moderately higher in the medial vestibular nucleus compared to the superior, lateral and spinal nucleus after CM (Fig. 3c).

### TNC-projecting VN neurons were activated after CM

**Fig. 4:**
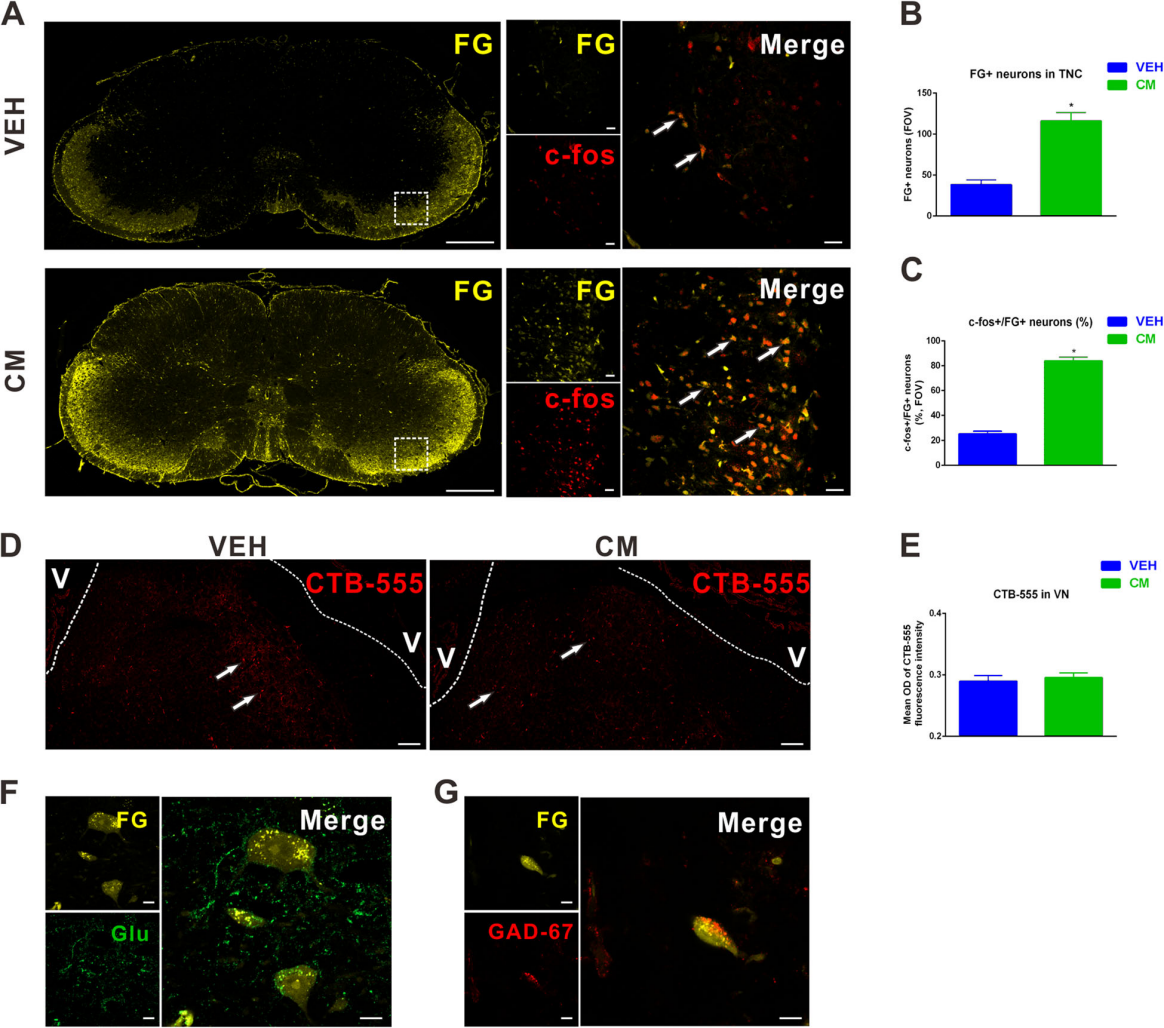
Trigeminal nucleus caudalis (TNC)-projecting vestibular nuclei (VN) neurons were activated after CM. **a** Representative images of FG (yellow) co-labeled with c-fos (red) neurons was accumulated predominantly in the superficial layer of the TNC. Quantitative assessments of FG+ neurons (**b**) and c-fos+/FG+ neurons (**c**) were significantly increased in the TNC compared with the vehicle group. FOV = 1.02 × 10^6^ μm^3^. **d**, **e** Representative images of immunofluorescence staining and quantification showing the distribution of retrogradely CTB-555 (red) labeled neurons in the VN. **f** Representative images showing three glutamate (green) immunofluorescent and FG (yellow)-immunolabeled globular, multipolar and fusiform neurons in the TNC projecting to the VN. **g** Representative images showing a GABA (red) immunofluorescent and FG (yellow)-immunolabeled elliptical neuron in the TNC projecting to the VN. (**a**: left row) Scale bar = 1 mm, (**a**: right row) Scale bar = 50 μm, **d** Scale bar = 100 μm, **f**, **g** Scale bar = 5 μm; *P < 0.05 compared with vehicle, n = 8/group. Data are mean ± SEM. Student’s *t*-test. V: ventricle

To determine whether trigeminovestibular neurons are activated by CM, we evaluated c-fos+/FG+ neurons in the TNC, as well as CTB-555+ neurons in the VN after CM. Retrogradely transported FG was accumulated under physiological condition predominantly in the superficial layer of the TNC (Fig. 4a). Compared to the vehicle group, the number of FG+ neurons increased significantly in the bilateral TNC after CM (Fig. 4a and b). We also found that the number of c-fos+/FG+ neurons were significantly increased in rats with CM compared to the vehicle group (vehicle vs. CM = 25.3 ± 2.2 vs. 83.9 ± 3.0), revealing that FG+ neurons in the TNC were primarily CM-activated neurons (Fig. 4a-c). In the meantime, CTB-555+ neurons dispersed throughout the VN (Fig. 4d). There was a slight increase in CTB-555 immunoreactivity in the CM group compared to vehicle group, which was not reach statistically difference (Fig. 4d-e).

We further sought to localize the amino acid in the cells that were both activated after CM and had direct projections to the VN. We performed immunofluorescence concomitantly for FG, glutamate and GABA. CM-activated TNC neurons with projections to VN were either glutamate- or GABA- immunofluorescent (Fig. 4f and g), suggesting both of excitatory and inhibitory input to these trigeminovestibular neurons. Aside from neurotransmitter content, the glutamate-immunofluorescent activated projection neurons were cytologically indistinguishable from the GABA immunofluorescent subpopulation (Fig. 4f and g).

### Knockdown of CGRP in the trigeminal ganglion reduced c-fos and CGRP levels in the TNC and VN after CM

**Fig. 5:**
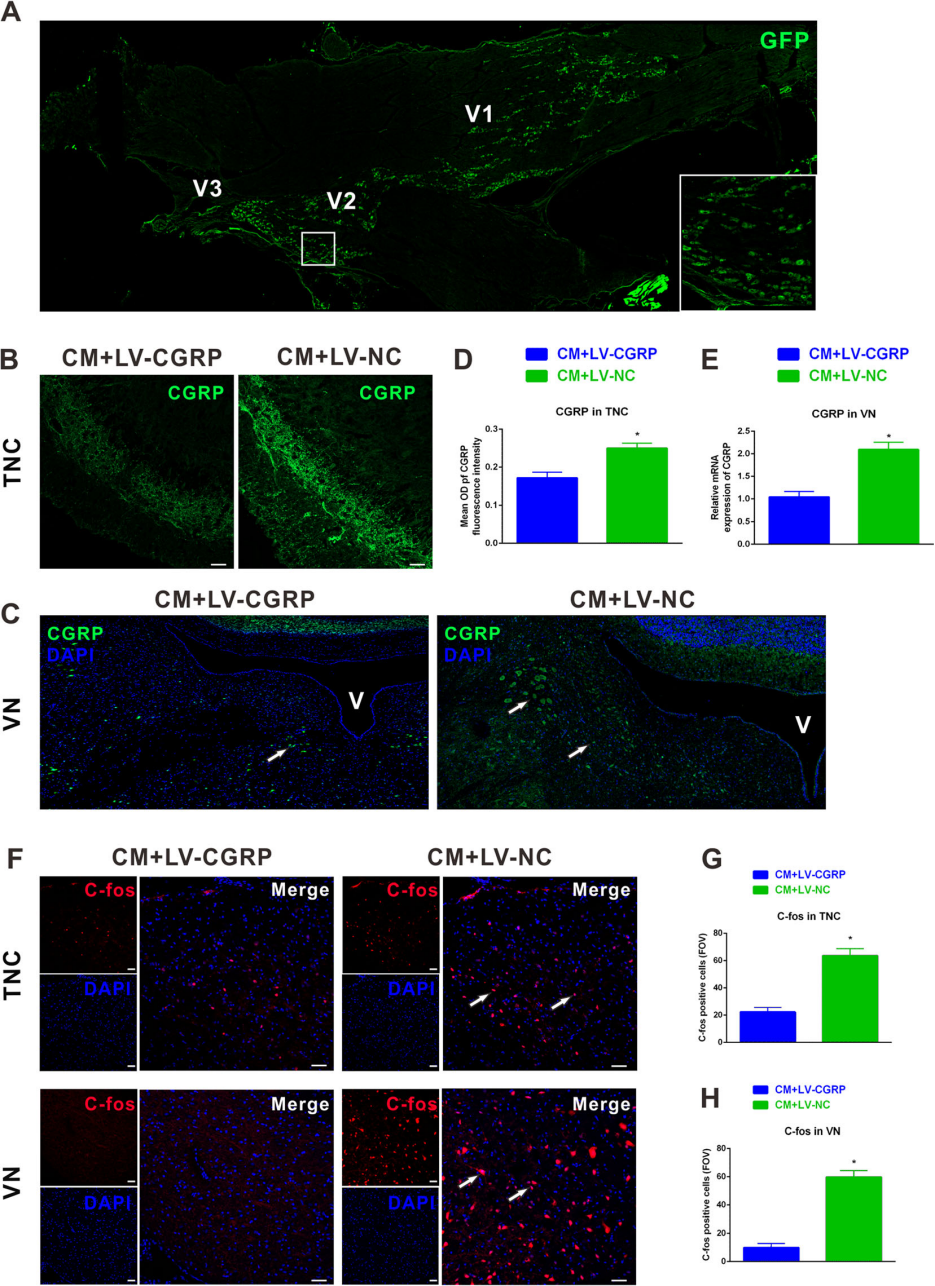
Knockdown of calcitonin gene-related peptide (CGRP) in the trigeminal ganglion reduced c-fos and CGRP levels in the trigeminal nucleus caudalis (TNC) and vestibular nuclei (VN) after CM. **a** Representative images of immunofluorescence staining showing GFP (green) labeled lentivirus was injected into the trigeminal ganglion and dispersed in V1 and V2 branches. Representative images of immunofluorescence staining showing the immunoreactivity of CGRP in the TNC (**b**) and VN (**c**). Quantitative assessments showed that pretreatment of lentiviral vectors comprising CGRP short hairpin RNA (LV-CGRP) significantly reduced CGRP immunoreactivity in the TNC (**d**) and mRNA levels of CGRP in the VN (**e**). **f** Representative images showing the c-fos + neurons in the TNC and VN. (**g**) In the LV-CGRP treated group, the number of c-fos positively stained neurons in the TNC and VN significantly reduced compared with blank lentiviral vectors (LV-NC). FOV = 1.02 × 10^6^ μm^3^. **b**, **f** Scale bar = 50 μm; *P < 0.05 compared with vehicle, n = 8/group. Data are mean ± SEM. Student’s *t*-test

In vivo studies suggest that CGRP is synthetized by small- and medium-diameter neurons in the trigeminal ganglion and released from their central terminals in the brainstem to produce the central sensitization in CM [15]. To investigate the effects of CGRP knockdown on neuronal activation in the VN after CM, we injected lentiviral vectors comprising CGRP short hairpin RNA (LV-CGRP) or blank lentiviral vectors (LV-NC) with enhanced green fluorescence protein (GFP) into bilateral trigeminal ganglion 2 weeks before the initial NTG administration. Fluorescently labeled neurons were abundantly accumulated in the V1 and V2 branches of the trigeminal ganglion (Fig. 5a).

Compared with LV-NC, pretreatment of LV-CGRP in the trigeminal ganglion markedly attenuated CGRP immunoreactivity in the TNC after CM (Fig. 5b and d). The addition of LV-CGRP after CM significantly reduced CGRP mRNA levels in the VN (LV-CGRP vs. LV-NC = 1.0 ± 0.1 vs. 2.1 ± 0.2, Fig. 5c and e). In the meanwhile, c-fos immunoreactivity in the TNC (LV-CGRP vs. LV-NC = 22.4 ± 3.2 vs. 63.6 ± 5.1) and VN (LV-CGRP vs. LV-NC = 9.9 ± 3.0 vs. 60.0 ± 4.5) were both significantly reduced in the CM + LV-CGRP group compared to CM + LV-NC group (Fig. 5g-h).

### Knockdown of CGRP in the trigeminal ganglion ameliorated hyperalgesia and vestibular dysfunction after CM

**Fig. 6:**
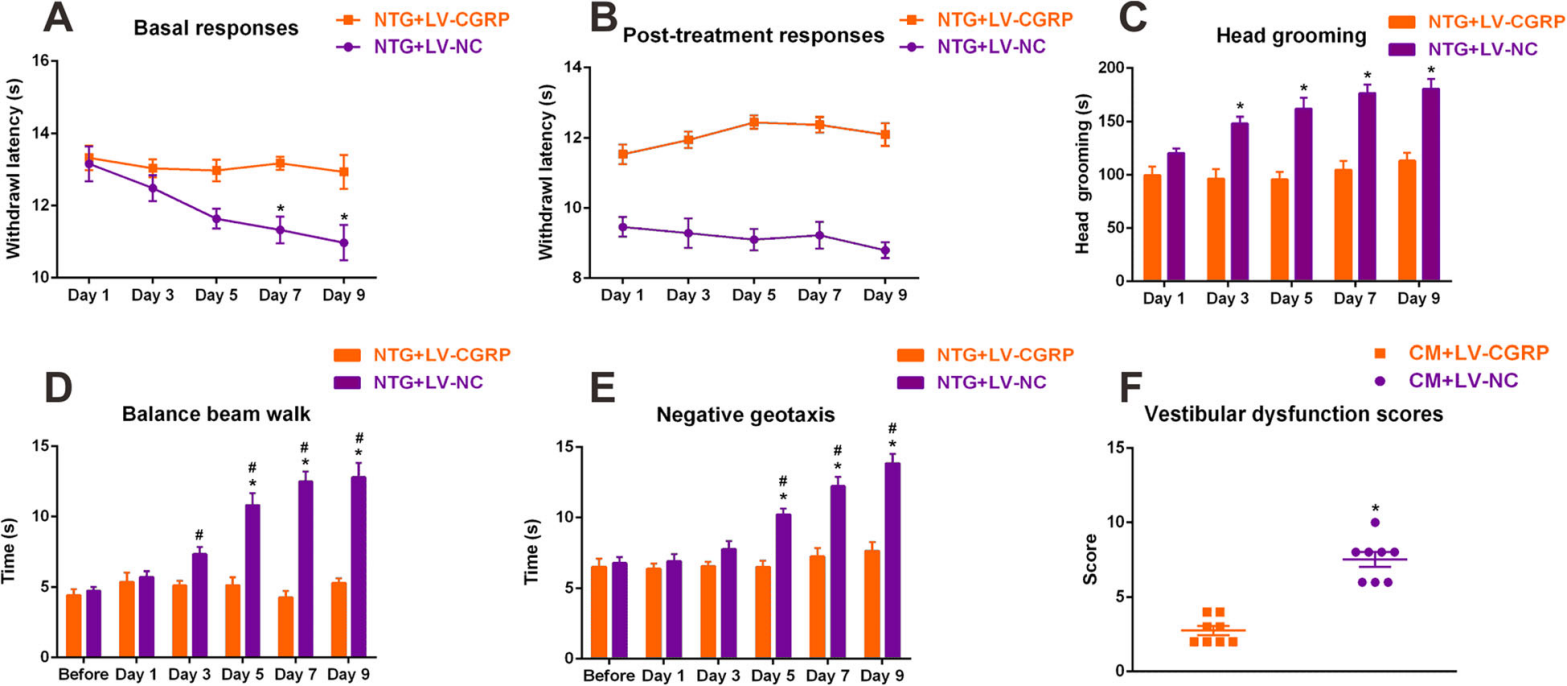
Knockdown of calcitonin gene-related peptide (CGRP) in the trigeminal ganglion ameliorated hyperalgesia and vestibular dysfunction after CM. **a** Basal and post-treatment responses (**b**) of hindpaw thermal hyperalgesia were significantly decreased with pretreatment of LV-VGRP. (**c**) Head grooming time was significantly decreased in LV-CGRP-treated rats compared with LV-NC-treated group. Balance beam walk (**d**), geotaxis reflex (**e**) and vestibular dysfunction scores (**f**) showing that pretreatment of LV-CGRP restored impairments in dynamic and static postural control compared with the LV-NC group. *n* = 8/group. Data are mean ± SEM. Analysis of variance (ANOVA), Tukey; *p < 0.05 compared with vehicle, #*p* < 0.05 compared with before NTG injection

To further clarify the effects of CGRP knockdown on CM-induced hyperalgesia and vestibular dysfunction, a series of behavior studies were assessed. The basal and post-treatment thermal thresholds were significantly increased in LV-CGRP-treated group compared with those in the LV-NC group (Fig. 6a and b), and significant changes were observed on day 7, and on day 9. LV-CGRP-treated rats spent less time on head grooming compared to the LV-NC group since day 3, indicating a markable improvement on spontaneous facial pain (Fig. 6c). In the balance beam walk and negative geotaxis test, LV-CGRP-treated animals spent less time on traversing beam and flipping to the prone position (Fig. 6d and e). Moreover, LV-CGRP-treated rats performed significantly better in dynamic postural controls compared with LV-NC group, as demonstrated by the less vestibular dysfunction score (Fig. 6f). Collectively, knockdown of CGRP in the trigeminal ganglion attenuated neuronal activation and CGRP translation in the VN and restored vestibular function after CM, indicating a key role of CGRP in the development of vestibular dysfunction in CM.

## Discussion

Vestibular symptoms are prevalent among patients with migraine [2, 3]. The mechanism that relate vestibular symptoms to migraine had not been well elucidated. Thus, current therapies are based on experts’ experience and little clinical progress has been made to develop therapeutic strategies targeting this subpopulation. Central sensitization has been demonstrated as a primary pathophysiological process after CM, in which CGRP has an essential role [6]. To date, clinical trials witness great success in anti-CGRP treatment on migraine headache [45]. Therefore, whether sensitization in the vestibular nucleus attribute to the development of vestibular dysfunction after CM, and whether anti-CGRP treatment could attenuate vestibular symptoms in migraine needs further illustrating.

The present study revealed that the development of vestibular dysfunction coincide with that of hyperalgesia in a rat model of CM. Meanwhile, the structure of vestibular afferent terminals was preserved after CM. CM induced neuronal activation in the TNC and VN, and CM-activated neurons in the TNC were primarily TNC-projecting VN neurons. In vivo knockdown of CGRP in the trigeminal ganglion could alleviate neuronal activation and upregulation of CGRP in the VN, further attenuating vestibular dysfunction in CM rats. Collectively, these studies suggested the possibility of vestibular sensitization to impair vestibular function after CM, and anti-CGRP treatment to restore vestibular dysfunction in patients with CM.

Systemic recurrent NTG administration has been proven to be a reliable method to produce the preclinical CM model: inducing acute and sustained hypersensitivity, which mimics the core clinical characteristics of CM; causing distinctively associated features, like photophobia, facial grimace behaviors, and upregulation of CGRP in the TNC and dura mater [15, 45]. However, little is known about the changes of vestibular-mediated behaviors in this CM model. Further, since up to one-fourth percentage of peripheral vestibular deficits, like hearing loss and endolymphatic hydrops, has been detected among migraine patients with vestibular symptoms and the development of vestibular symptoms often lagged several years behind headache [8, 46], some researchers deduce that the vestibular dysfunction in migraine patients may result from the damage of peripheral vestibular apparatus [40–42, 47]. In the present study, we first revealed that recurrent NTG administration produced allodynia and significant vestibular dysfunction, and the severity of vestibular dysfunction was comparable with kainic acid treated group. The development of vestibular dysfunction in our study is in line with clinical-based studies showing that most migraine patients are vestibular symptoms-free during the initial years of their migraine experience, but gradually develop motion intolerance or vestibular symptoms with increasing frequency of migraine attacks [9]. Meanwhile, morphological examination of the afferent terminal impairments showed that the swelling and structure disorders were much less pronounced in the CM group when compared with kainic acid treated group. These data indicated that it might be more likely a central, rather peripheral, component contribute to the vestibular dysfunction in migraine.

Migraine has been more generally considered as a neurological disorder of sensitization, with heightened sensitivity to light, sound, smell and motion [48]. Clinical and preclinical studies show that the central sensory systems are sensitized in migraine [49]. Thus, a reasonable hypothesis is that sensitization of vestibular pathways may contribute to the enhanced motion sickness susceptibility and episodic attacks of vestibular symptoms in migraine [50]. C-Fos has been widely used as an early indicator of neuronal activation that normally responds within 2 h [51]. Long-term c-Fos expression after NTG administration suggests continuous activation of secondary sensory neurons [37, 52]. In our study, significant fos-protein labeling in the superficial lamina of the TNC and VN after chronic NTG administration were observed. It is noteworthy that anatomic pathways giving extensive projections between brainstem regions associated with the TNC and the VN have been traced in the physiological condition [12]. We then asked whether CM-activated neurons belonged to the population of trigeminovestibular neurons, and whether persistent activation of TNC neurons facilitated subsequent activation of VN neurons. To gain a further understanding, FluoroGold and CTB-555 were selected as retrograde tracers in vehicle and CM groups, mainly based on published reports of their sensitivity and uni-directional transportation [22, 53]. Our immunofluorescent results showed that FluoroGold labeling neurons were predominantly located in the superlayer of the TNC, and CM-activated neurons in the TNC were primarily TNC-projecting VN neurons. Furthermore, to investigate whether changes observed in the VN were due to the hypersensitivity of TNC neurons or directly caused by NTG, viral vectors containing CGRP RNAi were injected into the trigeminal ganglion to knockdown CGRP synthesis in NTG-treated animals. We observed that c-fos expression was significantly decreased in the TNC among CM group, and similar pattern was also observed in the VN. Above data indicated the possibility of trigeminal-mediated sensitization of vestibular nucleus neurons in migraine.

CGRP is a 37-amino acid neuropeptide, and widely expressed in central and peripheral nervous system, with prominent localization in the outer laminae of the spinal cord dorsal horn and TNC [15, 54]. The majority of CGRP in the TNC is synthesized in small- and medium-diameter neurons of trigeminal ganglion, and transported to central terminals [15]. CGRP is believed to be a key regulator in central sensitization of trigeminovascular neurons, attributing to migraine headache and associated hypersensitivity [6, 15]. Contrast to a nociceptive role, CGRP enhances the abnormal pain sensitivity rather than normal acute nociceptive signals, as evidenced by the fact that intrathecal administration of CGRP did not alter response thresholds to noxious thermal stimuli in normal rats, while anti-CGRP antiserum significantly blocked hyperalgesia under inflammatory condition [55]. Previous study demonstrates that the CGRP protein is expressed in afferent terminals in the TNC [15], and in the vestibular nuclei and cerebellum, CGRP immunoreactivity can be detected on neuronal somas [54, 56]. In consistent with previous study, we found the extensive expression of CGRP immunoreactive fibres in the superlayer of the TNC and CGRP-positive neurons in the VN [37, 56]. To date, little evidence shows the change of CGRP expression in the VN after CM. In this study, we noted that CGRP expression was significantly elevated in the VN after CM, suggesting that the endogenous CGRP expression change in the VN was in response to CM. We also found that CGRP positively stained neurons could be detected in four major vestibular nuclei after CM, with a slight more expression in medial vestibular nucleus according to the rat brain atlas of Paxinos and Watson [56]. The medial vestibular nucleus is the largest nucleus within the vestibular complex and has various types of neurons [57]. Medial vestibular nucleus neurons are essential to the maintenance of vestibulo-ocular reflex, especially in stabilizing the images during head movement [58]. Clinical-based studies provide supporting evidence that ocular and perceptual thresholds are significantly increased in migraine patients with vestibular symptoms relative to those without vestibular symptoms, implying that sensitization of the medial vestibular nucleus might be a primary pathophysiological process underlying the vestibular hypersensitivity in migraine patients with vestibular symptoms [10, 11].

Clinical trials investigating CGRP antibodies or CGRP receptor antagonists have shown statistically significantly efficacy for migraine treatment [45]. Both of CGRP antibodies or CGRP receptor antagonists have limited permeability of blood-brain barrier [59], indicating that trigeminal ganglion may be the target region for anti-CGRP therapy in migraine headache [59]. Present study used lentiviral vectors comprising CGRP short hairpin RNA (LV-CGRP) to knockdown CGRP production in the trigeminal ganglion. The neuronal activation and CGRP expression in the TNC were decreased, meanwhile, the migraine-associated hyperalgesia and spontaneous facial pain were alleviated after CM, suggesting knockdown of CGRP in the trigeminal ganglion could efficiently block development of sensitization of the TNC and relieve migraine headache after CM. We also found that knockdown of CGRP in the trigeminal ganglion had similar effects on the VN, and restored vestibular dysfunction after CM. Currently, the relationship between CGRP and sensitization of VN neurons remains obscure. Indirect evidence shows that CGRP expression is significantly increased among VN neurons in a preclinical model of motion sickness, whilst anisodamine could decrease CGRP expression in the VN and relieve motion sickness [17]. Collectively, these data pointed a role of CGRP in the VN to facilitate sensitization of VN neurons, and blocking CGRP in the trigeminal ganglion might relieve vestibular symptoms in migraine.

In this study, we focused on c-fos and CGRP expression changes in the TNC and VN, as well as the behavior changes after CM or CGRP knockdown. Previous studies showed that the indicators of central sensitization also contained electrophysiologic, like spontaneous and evoked activity of wide-dynamic-range neurons or response thresholds [15]. Hence, whether CM could induce electrophysiological changes in the VN neurons remains to be determined. Moreover, immunohistochemistry study reported that CGRP could be detected in various brain regions, including cerebral cortex and thalamic nuclei [54], thus, the effects of other brain regions on the regulation of neuronal activation and CGRP expression in the VN warrant further investigation.

## Conclusions

In conclusion, we demonstrated the sensitization of vestibular nucleus neurons in a preclinical model of CM, and down-regulation of CGRP after CM could improve vestibular dysfunction, suppress neuronal activation, and reduce CGRP expression in the VN. Therefore, anti-CGRP might be a promising treatment strategy for ameliorating vestibular dysfunction in migraine patients with vestibular symptoms.

## Data Availability

The data used and analyzed in this article are available from on reasonable request.

## Abbreviations

CGRP: Calcitonin gene-related peptide
CM: Chronic migraine
FG: Fluorogold
FOV: Field of view
GFP: Green fluorescent protein
LV-CGRP: Lentiviral vectors comprising CGRP short hairpin RNA
LV-NC: Lentivirus vector negative control
NTG: Nitroglycerin
OD: Optical density
TNC: Trigeminal nucleus caudalis
VEH: Vehicle
VM: Vestibular migraine
